# Active tuberculosis patients have high systemic IgG levels and B-cell fingerprinting, characterized by a reduced capacity to produce IFN-γ or IL-10 as a response to *M.tb* antigens

**DOI:** 10.3389/fimmu.2023.1263458

**Published:** 2023-10-26

**Authors:** Julio Flores-Gonzalez, Alexia Urbán-Solano, Lucero A. Ramón-Luing, Juan Carlos Cancino-Diaz, Araceli Contreras-Rodriguez, Everardo Curiel-Quesada, Rogelio Hernández-Pando, Leslie Chavez-Galan

**Affiliations:** ^1^ Laboratory of Integrative Immunology, Instituto Nacional de Enfermedades Respiratorias Ismael Cosio Villegas, Mexico City, Mexico; ^2^ Department of Microbiology, Laboratory of Immunomicrobiology, Escuela Nacional de Ciencias Biológicas, Instituto Politécnico Nacional, Mexico City, Mexico; ^3^ Department of Biochemistry, Escuela Nacional de Ciencias Biológicas, Instituto Politécnico Nacional, Mexico City, Mexico; ^4^ Department of Pathology, Section of Experimental Pathology, Instituto Nacional de Ciencias Médicas y Nutrición Salvador Zubirán, Mexico City, Mexico

**Keywords:** tuberculosis, IFN-g, IL-10, B cells, humoral immunity, flow cytometry

## Abstract

**Introduction:**

Tuberculosis (TB) is a bacterial infection caused by *Mycobacterium tuberculosis* (*M.tb*). B cells are the central mediator of the humoral response; they are responsible for producing antibodies in addition to mediating other functions. The role of the cellular response during the TB spectrum by B cells is still controversial.

**Methods:**

In this study, we evaluated the distribution of the circulating B cell subsets in patients with active and latent TB (ATB and LTB, respectively) and how they respond to stimuli of protein or lipid from *M.tb.*

**Results:**

Here, we show that ATB patients show an immune fingerprinting. However, patients with drug-sensitive- (DS-TB) or drug-resistant- (DR-TB) TB have altered frequencies of circulating B cells. DS-TB and DR-TB display a unique profile characterized by high systemic levels of IFN-γ, IL-10, IgG, IgG/IgM ratio, and total B cells. Moreover, B cells from DR-TB are less efficient in producing IL-10, and both DS-TB and DR-TB produce less IFN-γ in response to *M.tb* antigens.

**Conclusion:**

These results provide new insights into the population dynamics of the cellular immune response by B cells against *M.tb* and suggest a fingerprinting to characterize the B-cell response on DR-TB.

## 1 Introduction

Tuberculosis (TB) is one of the deadliest infectious diseases worldwide, and its etiological agent is the bacilli *Mycobacterium tuberculosis* (*M.tb*). Clinically, TB could be divided into latent TB (LTB) or active TB (ATB); LTB patients are infected with *M.tb* but remain asymptomatic, and although it has been estimated that one-fourth of the world’s population have LTB, the immune mechanism to maintain the latent status of TB remains poorly understood (1).

In recent years, an increase in ATB with drug-resistant strains (DR-TB) has been reported; the World Health Organization estimated 450,000 cases of DR-TB in 2021, representing an increase of 3.1% compared with 2020 (2). To eradicate TB, it is necessary to identify LTB and ATB patients and to establish the immunological differences in the infection chronicity, which could be fundamental to identifying new diagnostic markers or treatment schemes.

The infection chronicity by *M.tb* induces immunological changes that could be effective biomarkers. Some evidence indicates that compared with LTB or drug-sensitive TB (DS-TB), DR-TB patients have increased specific subgroups of monocytes and T cells sustained even after administering anti-TB drugs (3). By contrast, the B-cell response has been little explored in the context of DR-TB and LTB. Recently, using antigen-specific TCR-transgenic mice, it has been reported that *M.tb* infection remodels the lymph nodes’ architecture by increasing the number and paracortical translocation of B cells to impair the naive CD4^+^ T-cell activation (4).

The B-cell functions described more frequently are antibody production, phagocytosis, antibody-dependent cellular cytotoxicity, and complement-mediated lysis of pathogens or infected cells (5). Although the germinal reaction is a critical part of the antigen-specific antibody production by antigen-specific B cells, it is known that B cells can also produce cytokines in response to stimulation *in vivo* or *in vitro* (6); these cells can detect pathogen-associated molecular patterns (PAMPs) through general pattern recognition receptors (PRRs) (7). B cells produce lower cytokine levels than T cells and myeloid cells when they are stimulated with *M.tb* antigens (8); some of the more antigenic are the protein PPD (purified-protein derivate) and lipids such as PIM (phosphatidyl-inositol mannosides) and LAM (lipoarabinomannan) (9). Studies *in vitro* have shown that *M.tb* glycolipids induce a CD4^+^ T helper type 1 (TH1) polarization and increase the frequency of IL-10-producing B cells (10). In this regard, it has been identified that the presence of another IFN-γ-producing B-cell subset requires the IFN-γR expression and the T-box transcription factor to amplify the TH1 polarization (11).

Additionally, data support that CD38 and CD23 expression on T cells is useful as a marker of infection resolution in TB (12, 13). However, its expression on B cells is unknown. CD38 is a type II transmembrane protein with both enzymatic and receptor functions, its extracellular domain catalyzes the conversion of nicotinamide adenine dinucleotide (NAD) to cyclic adenosine diphosphate ribose (cADPR), and it mediates processes such as cellular migration, phagocytosis, and antigen presentation or cytokine release (14).

In the TB context, CD38^+^ cell populations are increased in ATB (15). Recently, these populations have been associated as a biomarker for diagnosing and clinically monitoring TB (12, 16). Moreover, CD38 is used as a marker to classify circulatory naive B cells into resting (CD38^+^) or activated (CD38^−^), whereas the subsets of mature human B cells induce CD38 upon activation (17). On the other hand, CD23 is a receptor that negatively regulates B-cell receptor signaling (18). It has been reported that unswitched B cells (CD23^−^) are decreased in ATB but increase post-treatment compared with other lung diseases, suggesting their potential utility as TB treatment response biomarkers (19, 20), but the CD23 expression on B cells during DR-TB has been little explored.

Current knowledge highlights the role of B cells during TB; it still needs to be determined if B cells have altered phenotypes and functions that allow discrimination between ATB and LTB or if the resistance to drugs also impacts the B-cell subsets. Therefore, understanding CD38 and CD23 dynamics expression is essential to clarifying the role of B cells in maintaining the inflammatory microenvironment in TB. Here, we evaluated the B-cell subsets in household contacts of TB patients (HC), LTB subjects, and DS-TB and DR-TB patients by phenotype and capacity to produce IFN-γ and IL-10 in response to lipid and protein stimuli, and finally, correlations between B-cell subsets and clinical characteristics were identified.

## 2 Materials and methods

### 2.1 Ethics statement

This study was approved by the Institutional Ethics Committee of the Instituto Nacional de Enfermedades Respiratorias Ismael Cosío Villegas (Protocol numbers B04-15 and B01-22) at Mexico City. All participants signed a written informed consent. All procedures were performed in agreement with the 1964 Helsinki Declaration and the ethical standards of the Institutional Ethics Committees.

### 2.2 Study population

Thirty pulmonary TB patients diagnosed at the Tuberculosis Outpatient Clinic, INER, Mexico, were enrolled. Fifteen patients were diagnosed with DS-TB, and 15 were drug-resistant (DR-TB). The DS- or DR-TB diagnosis was by PCR Xpert-MTB/RIF (Cepheid, CA, United States). In addition, drug sensitivity testing to first- and second-line anti-TB drugs was evaluated using the BD BACTEC™ MGIT™ systems. The results of these assays were confirmed using a Lowenstein–Jensen (LJ) medium.

**Figure 1:**
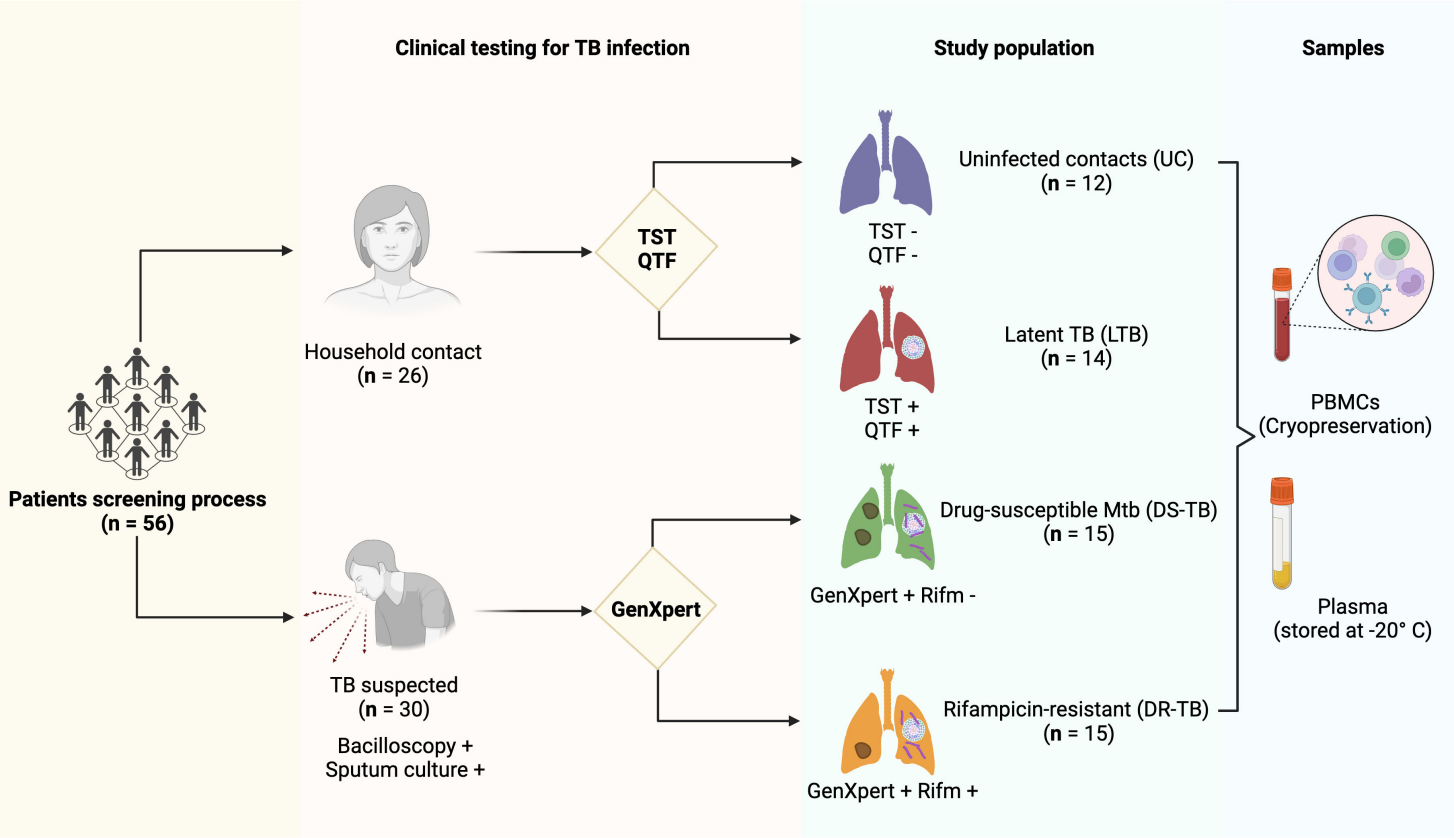
Patient enrollment and testing workflow. A total of 56 participants were enrolled: 26 were household contacts, and 30 had a suspected TB diagnosis confirmed by bacilloscopic and sputum culture testing. The household contact group was divided according to PPD and QTF testing on 12 uninfected contacts (UC) with negative TST and QTF testing and 14 latent TB subjects (LTB) with positive TST and QTF testing. In addition, the TB suspected group was divided based on GenXpert testing: 15 drug-susceptible *M.tb* (DS-TB) with negative results to rifampicin-resistant gen testing and 15 rifampicin-resistant (DR-TB). In each group, PBMCs and plasma samples were stored. TB, tuberculosis; TST, tuberculin skin test; QTF, QuantiFERON®-TB Gold Plus; GenXpert, PCR Xpert-MTB/RIF; PBMCs, human peripheral blood mononuclear cells. The figure was created in BioRender.

A group called HC was defined as individuals who shared the same enclosed living space for one or more nights a week or extended periods during the day with the TB patient 3 months before treatment began. QuantiFERON®-TB Gold Plus (QFT®-Plus) test (Qiagen, Hilden, Germany) and tuberculin skin test (TST) were evaluated in HC, and from them and according to test results, LTB was diagnosed. Among the HC group, 12 subjects negative to QFT and TST were considered uninfected contact (UC), and 14 positives to QFT and TST were considered LTB (**Figure 1**). After the diagnosis, patients received the corresponding treatment following the international normative and a clinical follow-up. Clinical, laboratory, and radiological data were obtained from the medical records of all ATB patients.

Individuals with comorbidities such as human immunodeficiency virus (HIV), pulmonary cancer, chronic obstructive pulmonary disease, or autoimmune conditions and solid-organ transplant recipients were excluded from this study.

### 2.3 Sample collection

Ten milliliters of heparinized blood samples were collected from DS-TB and DR-TB patients, LTB subjects, and UC. Then, peripheral blood mononuclear cells (PBMCs) were isolated by density gradient (Lymphoprep™). The trypan blue (Gibco™) exclusion assay was used to determine the number of viable cells, and a minimum of 85% viability was required to be able to process the cells. Plasma was stored at −20°C until use.

### 2.4 B-cell phenotyping and high-dimensional data analysis

PBMCs (1 × 10^6^) per ml of UC (*n* = 8), LTB (*n* = 10), DS-TB (*n* = 10), and DR-TB (*n* = 10) were stained with monoclonal antibodies (mAbs) against CD3, CD19, IgG, IgM, IgD, and CD38 (BioLegend, San Diego, CA, USA). Fluorescence minus one (FMO) condition was stained and acquired in parallel to identify background levels of staining. Dead cells were omitted using viability staining Zombie Red Dye solution (BioLegend). More details of the antibodies used can be found in **Table S1**.

The data were acquired using a FACS Aria II flow cytometer (BD Biosciences, San Jose, CA, USA) equipped with the FACSDiva 6.1.3 software (BD Biosciences). In each condition, at least 50,000 events were acquired per sample. The flow cytometry data file (FCS) was analyzed using FlowJo (FlowJo)™ v10.6.1.

Gating was performed doing flowAI analysis (R package flowAI, version 1.28.0) as a quality control analysis to detect and remove anomalies based on the flow rate, signal acquisition, and dynamic range in each FCS data and select good events (**Figure S1A**). Next, each FSC data was limited to singlet events through forward scatter (FSC-A vs. FSC-H), and viability plots were selected (negative events to viability marker). Subsequent PBMCs were chosen through side scatter and forward scatter (FSC-A vs. SSC-A), and a second singlet events plot was made through side-scatter area versus height (SSC-A vs. SSC-H).

B cells were identified as CD19^+^ events on each FSC data (**Figure S1B**). From all CD19^+^ samples, a concatenated data file was generated (**Figure S1C**). Subsequently, unsupervised clustering was performed on the expression values of the lineage markers using the FlowSOM algorithm (R package FlowSOM, version 1.14.1), which uses a self-organizing map followed by hierarchical consensus metaclustering to detect eight metaclusters (**Figure S1D**).

The analysis was made as recommended in the reference protocol (21). Subsequently, uniform manifold approximation and projection (UMAP) (R package UMAP, version 0.2.0.0) was performed on IgD, IgM, IgG, and CD38 expression. The FlowSOM populations were overlayed onto the UMAP projection. Finally, the cells were colored according to their FlowSOM cluster membership (**Figure S1E**).

For plasma B-cell identification and characterization, 1 × 10^6^ PBMCs/ml of HC (*n* = 8), LTB (*n* = 10), DS-TB (*n* = 10), and DR-TB (*n* = 10) were stained with mAbs against CD3, CD19, CD20, CD5, CD10, and CD1d (BioLegend, San Diego, CA, USA). We included FMO and flowAI analysis in parallel to identify background levels of staining; dead cells were omitted using viability staining. More details of the antibodies used can be found in **Table S1**.

### 2.5 PBMC culture and stimulation

PBMCs (5 × 10^5^) per ml of UC (*n* = 4), LTB (*n* = 5), DS-TB (*n* = 5), and DR-TB (*n* = 5) were cultured in a 24-well plate (Corning, New York, NY, USA) with Roswell Park Memorial Institute (RPMI-1640, Gibco™) medium supplemented with 2 mM of L-glutamine (Gibco™), 1 M of 4-(2-hydroxyethyl)-1-piperazineethanesulfonic acid (HEPES, Gibco™), penicillin–streptomycin–amphotericin B (Gibco™), and 10% fetal bovine serum (Gibco™).

PBMCs were stimulated with 10 mg/mL of proteins (NR-14831) or lipids (NR-14837) from the cell membrane of *M.tb*, strain H37Rv provided by BEI Resources. The culture was maintained at 37°C, 5% CO_2_, 72 h. Unstimulated PBMCs (only culture medium) were included as a negative control.

### 2.6 Intracellular cytokine evaluation

Four hours before the end of the culture, 1 μg/mL of brefeldin A (bfA) (GolgiStop™, BD Biosciences) was added to accumulate intracellularly trapped cytokines. At the end of the culture, PBMCs were recovered and stained with mAbs against CD3, CD19, IgM, CD23, and CD27 (BioLegend) for 30 min at 4°C. Then, cells were washed with Cell Staining Buffer (BioLegend), permeabilized with BD Cytofix/Cytoperm™ buffer (BD Biosciences), and incubated for 20 min at 4°C. Hereafter, cells were washed and incubated for 30 min at 4°C with mAbs against IL-10 and IFN-γ (BioLegend). More details of the antibodies used can be found in **Table S1**. Acquisition, processing, and analysis of data were performed as described for B-cell phenotyping above.

The data were acquired using a FACS Aria II flow cytometer with the FACSDiva 6.1.3 software (BD Biosciences). In each condition, at least 50,000 events were acquired per sample. The flow cytometry data file (FCS) was analyzed using FlowJo, v10.6.1 (FlowJo™).

### 2.7 Enzyme-linked immunosorbent assay

Soluble plasma levels of IFN-γ (BioLegend), IL-10 (BioLegend), IgM (Invitrogen, Massachusetts, Estados Unidos), and IgG (Invitrogen, Massachusetts, Estados Unidos) were measured by enzyme-linked immunosorbent assay (ELISA) following the manufacturer’s recommendations (**Table S1**).

All proteins were quantified by comparison with the corresponding standard curve, and the optical density was measured using a microplate reader spectrophotometer (iMark, Bio-Rad, CA, United States) set to 450 nm.

### 2.8 Principal component and heatmap analyses

Serological data from patients tested for IFN-γ and IL-10 levels, IgM and IgG antibody levels, and B-cell phenotypes reported by flow cytometry were pooled into Microsoft Excel and imported to ClustVis (https://biit.cs.ut.ee/clustvis/). Original values were ln(x)-transformed without scaling applied to rows. Principal components were calculated using NIPALS PCA. *X*- and *Y*-axes show principal component 1 and principal component 2, which explain 38.6% and 23.3% of the total variance, respectively. Prediction ellipses are such that with a probability of 0.95, a new observation from the same group would fall inside the ellipse. *N* = 38 data points, grouped with a 95% confidence interval.

ClustVis was also used to generate a heatmap clustering based on serological and flow cytometry data from the 38 patient samples. Values were centered by subtracting the average soluble molecule and cell population from all samples from each data point.

### 2.9 Statistical analysis

The D’Agostino–Pearson test was used to test the normality of data. Non-normally distributed variables are shown as median value and interquartile range (IQR, 25–75). Kruskal–Wallis test with Dunnett’s post-test was used for multiple comparisons. *p*-values <0.05 were considered statistically significant (GraphPad software).

The Spearman correlation matrix was produced in R version 4.2.0 using the corrplot version 0.92 package. The color and size of the circles in the diagram represent the correlation’s magnitude and direction. The *p*-values were also calculated, and the data points with *p*-values larger than 0.05 are not shown in the diagram, indicating a lack of statistical confidence.

## 3 Results

### 3.1 Clinical characteristics

Demographic and clinical characteristics were compared between groups (**Table 1**). Overall, the study groups showed similar demographic characteristics, except that LTB was older [53 (44–59) years] than UC (*p* < 0.0247) and DS-TB (*p* < 0.0128). Gender was not different between groups; DS-TB patients had lower weight than UC (*p* < 0.0227) or LTB (*p* < 0.0124), and similarly, DR-TB patients had lower weight than UC (*p* < 0.0163) or LTB (*p* < 0.0147). In concordance, DS-TB and DR-TB showed lower BMI values [vs. UC (*p* < 0.0381) and LTB (*p* < 0.0110); UC (*p* < 0.023) and LTB (*p* < 0.0144), respectively]. Other clinical characteristics evaluated were not different.

**Table 1:** Participants’ characteristics.

Parameters	UC [A] (*n* = 8)	LTB [B] (*n* = 10)	DS-TB [C] (*n* = 10)	DR-TB [D] (*n* = 10)	*p*
**Age, years**	28 (21–52)	53 (44–59)	36 (24–41)	37 (26–61)	* [A vs. B] * [B vs. C]
**Male, *n* (%)^1^**	5 (63)	3 (30)	4 (40)	6 (60)	ns
**Female, *n* (%)^1^**	3 (38)	7 (70)	6 (60)	4 (40)	ns
**Weight, kg**	76 (68–81)	73 (64–83)	58 (49–65)	57 (52–62)	* [A vs. C] * [A vs. D] * [B vs. C] * [B vs. D]
**Height, m**	1.6 (1.5–1.7)	1.6 (1.5–1.6)	1.6 (1.5–1.7)	1.6 (1.6–1.7)	ns
**Body mass index, kg/m^2^**	28 (26–28)	32 (26–32)	21 (18–25)	22 (18–25)	* [A vs. C] * [A vs. D] * [B vs. C] * [B vs. D]
**TA systolic, mmHg**	110 (99–119)	119 (109–120)	108 (104–115)	107 (102–116)	ns
**TA diastolic, mmHg**	69 (61–74)	72 (70–77)	70 (69–78)	70 (65–73)	ns
**Heart rate, bpm**	68 (61–73)	71 (59–88)	74 (69–87)	91 (66–107)	ns
**Respiratory rate, bpm**	23 (19–24)	20 (18–24)	22 (21–24)	24 (21–25)	ns
**Temperature, °C**	36 (35.8–36.4)	36 (36.0–36.5)	36 (36.0–36.1)	36 (36.0–36.0)	ns
**Oxygen saturation, %**	94.5 (91.0–96.0)	94.5 (92.0–97.2)	96.0 (95.0–98.0)	94.0 (88.0–96.2)	ns

Data are represented with median and interquartile range except for sex, which is expressed as a percentage. The statistical comparison was performed using the Kruskal–Wallis test with Dunn’s multiple comparison test and ^1^chi-squared tests.

bpm, breaths per minute; ns, not significant.

*p < 0.05.

Regarding hematological parameters, the ATB groups (DS-TB and DR-TB) had an increased absolute number of leukocytes, platelets, neutrophils, and monocytes compared with UC and LTB (**Table 2**). No differences were observed concerning UC and LTB, except for neutrophil count, where UC subjects showed lower than LTB patients (*p* < 0.0106) (**Table 2**). Consistent with previous reports (22), DS-TB patients had increased platelet count (UC, *p* < 0.0376; LTB, *p* < 0.0376), and in addition, DS-TB had higher values than DR-TB (*p* < 0.0106). Contrarily, ATB groups showed a reduced hemoglobin and hematocrit concentration compared with UC and LTB subjects (**Table 2**).

**Table 2:** Laboratory data for hematological parameters.

Parameters	UC [A] (*n* = 8)	LTB [B] (*n* = 10)	DS-TB [C] (*n* = 10)	DR-TB [D] (*n* = 10)	*P*
**Leukocyte count, ×10^3^ cell/μL**	5.8 (4.2–7.1)	6.8 (5.2–8.3)	9.5 (8.3–12.6)	8.5 (6.0–10.3)	** [A vs. C] * [B vs. C]
**Lymphocyte count, ×10^3^ cell/μL**	2.1 (1.2–2.3)	1.8 (1.5–2.7)	1.6 (1.0–2.1)	1.6 (1.2–1.8)	Ns
**Monocyte count, ×10^3^ cell/μL**	0.4 (0.3–0.5)	0.4 (0.4–0.5)	0.6 (0.6–1.0)	0.6 (0.5–0.8)	* [A vs. C] * [A vs. D] * [B vs. C] * [B vs. D]
**Neutrophil count, ×10^3^ cell/μL**	2.6 (2.0–3.0)	4.0 (3.3–4.4)	6.2 (4.5–8.1)	4.6 (3.0–9.0)	* [A vs. B] *** [A vs. C] ** [A vs. D] * [B vs. C]
**Platelet count, ×10^3^ cell/μL**	232.0 (200.3–246.0)	225.5 (219.3–261.5)	482.5 (312.8–530.0)	279.5 (195.3–383.5)	** [A vs. C] * [B vs. C] * [C vs. D]
**Hemoglobin, g/dL**	15.9 (13.4–6.7)	14.4 (14.1–16.2)	12.8 (11.5–14.0)	11.5 (11.1–13.5)	* [A vs. C] ** [A vs. D] ** [B vs. C] * [B vs. D]
**Hematocrit, %**	48.1 (41.1–50.5)	44.6 (42.0–50.1)	37.8 (33.6–43.0)	36.1 (33.8–40.8)	** [A vs. C] ** [A vs. D] ** [B vs. C] ** [B vs. D]

Data are represented with median and interquartile range. The statistical comparison was performed using Kruskal–Wallis’s test with Dunn’s multiple comparison test.

ns, not significant.

*p < 0.05, **p < 0.01, ***p < 0.001.

According to biochemical parameters, only the level of albumin was lower in DR-TB than in HC (*p* < 0.0019) and LTB (*p* < 0.0030) (**Table 3**). ATB patients have typical TB disease clinical characteristics.

**Table 3:** Laboratory data for biochemistry parameters.

Parameters	UC [A] (*n* = 8)	LTB [B] (*n* = 10)	DS-TB [C] (*n* = 10)	DR-TB [D] (*n* = 10)	*p*
**Glucose, mg/dL**	98.0 (92.0–104.0)	103.0 (95.0–119.3)	103.5 (94.7–210.0)	122.0 (90.0–150.3)	ns
**Urea, mg/dL**	22.5 (15.5–34.0)	22.5 (17.1–31.0)	19.4 (16.0–22.4)	19.1 (15.0–25.5)	ns
**Blood urea nitrogen, mg/dL**	10.5 (7.2–16.0)	10.5 (8.0–14.5)	9.1 (7.5–10.5)	8.3 (7.0–11.1)	ns
**Uric acid, mg/dL**	5.5 (5.0–6.3)	5.6 (5.0–7.0)	5.7 (4.0–6.4)	5.0 (4.4–7.7)	ns
**Serum creatinine, mg/dL**	0.8 (0.7–1.0)	0.7 (0.7–0.9)	0.7 (0.6–0.8)	0.6 (0.5–0.9)	ns
**Total protein, g/dL**	7.1 (7.0–7.5)	7.3 (7.1–7.6)	7.5 (6.8–7.7)	7.1 (6.8–7.4)	ns
**Albumin, g/dL**	4.4 (4.1–4.4)	4.3 (4.1–4.4)	4.0 (2.5–4.1)	3.0 (3.0–3.5)	** [A vs. D] ** [B vs. D]
**Total bilirubin, mg/dL**	0.7 (0.5–0.9)	0.7 (0.6–1.1)	0.5 (0.5–0.6)	0.5 (0.4–0.8)	ns
**AST, IU/L**	28.0 (19.0–37.2)	24.5 (21.5–32.5)	26.5 (19.0–31.0)	23.5 (12.0–42.0)	ns
**LDH, IU/L**	155.5 (131.3–175.3)	137.0 (132.0–176.3)	148.0 (139.9–173.8)	147.8 (133.3–178.7)	ns
**ALP, IU/L**	72.5 (61.7–81.50)	78.5 (68.2–113.0)	84.0 (74.7–110.8)	82.5 (78.5–106.8)	ns
**HbA1c, %**	5.5 (5.3–6.0)	5.7 (5.5–6.0)	5.8 (5.5–8.0)	5.5 (5.0–8.5)	ns

Data are represented with median and interquartile range. The statistical comparison was performed using Kruskal–Wallis’s test with Dunn’s multiple comparison test.

ns, not significant.

**p < 0.01.

In summary, the study groups showed similar demographic characteristics. Although the LTB group was older than the others, we did not find changes in other parameters associated with age. LTB, in general, was homogeneous compared with UC. ATB groups (DS-TB and DR-TB) have typical TB disease clinical characteristics.

### 3.2 DR-TB patients show expansion and remodeling of the naive B-cell subset

**Figure 2:**
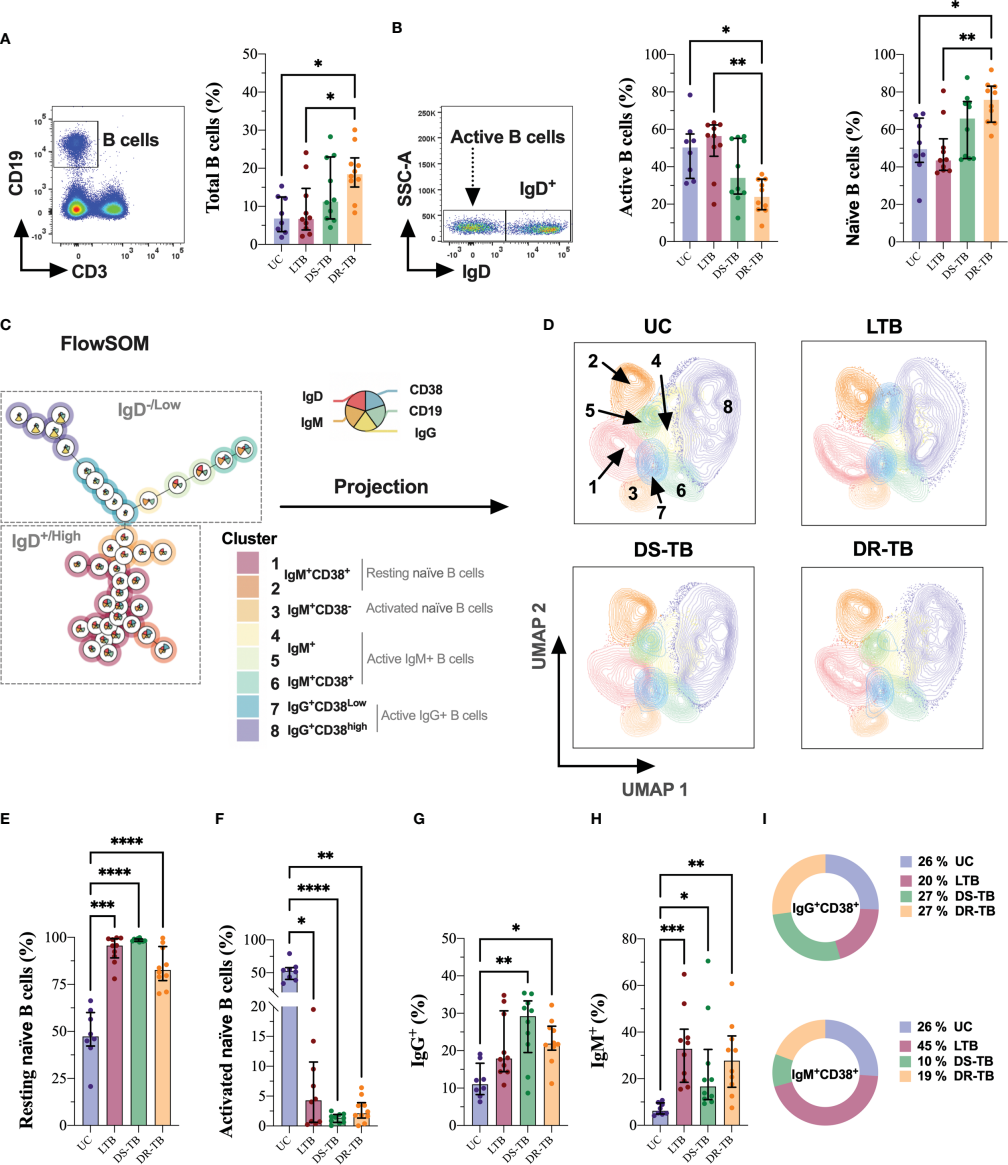
DR-TB showed increased B-cell frequency characterized by expansion of resting naive B cells. Frequency of B cells assessed in the study groups gated by the CD19 marker **(A)**. Based on this population, IgD was used to identify active and naive B cells **(B)**. FlowSOM **(C)** and its projection on UMAP **(D)** were used for the identification of B-cell subsets in multiparametric data sets identified as resting naive **(E)**, activated naive **(F)**, IgG^+^ **(G)**, IgM^+^ **(H)**, and activated status of IgG^+^ and IgM^+^ cells **(I)**. Data are represented as median and IQR values. Statistical comparisons were performed by the Kruskal–Wallis test. *p < 0.05, **p < 0.01, ***p < 0.001, and ****p < 0.0001.

The hematic biometry indicated leukocyte count alterations during TB (**Table 2**). However, B-cell count cannot be identified by this technique; using flow cytometry, naive (IgD^+^) or activated (IgD^−^) B-cell subpopulations were identified, as well as the CD38 expression (**Figure 2**).

B cells were identified based on CD19 expression, and our data showed that DR-TB had increased the frequency of total B cells compared with the UC and LTB groups (*p* < 0.0191 and *p* < 0.0408, respectively) (**Figure 2A**). However, DR-TB showed a lower frequency of active B cells compared with UC (*p* < 0.0318) and LTB (*p* < 0.0028), whereas the frequency of naive cells was higher than UC (*p* < 0.0423) and LTB (*p* < 0.0036) (**Figure 2B**).

Next, we performed the FlowSOM projection on UMAP dimensionality reduction on manually gated B cells, which clustered the population into eight islands (**Figures 2C, D**). FlowSOM and UMAP were used as tools for the identification of B-cell subsets in multiparametric data sets, and we identified eight metaclusters: metaclusters 1 and 2 corresponded to resting naive B cells; metacluster 3 was associated with a phenotype of activated naive B cells; metaclusters 4, 5, and 6 were related to active IgM^+^ B cells; and metaclusters 7 and 8 were associated with active IgG^+^ B cells.

Using the UMAP algorithm, cells were pooled according to the intensity of expression of each parameter, and they were visualized with color code from dark green (lowest expression) to dark red (highest expression) (**Figure S1E**). The spatial distribution of different B-cell subsets is modified (**Figure 2D**).

The frequency of B-cell subsets indicated that all TB groups have increased naive B cells in a resting state compared with UC (LTB, *p* < 0.0010; DS-TB, *p* < 0.0001; DR-TB, *p* < 0.0001, respectively) (**Figure 2E**). In comparison, activated naive B cells showed a significant decrease in all TB groups compared with UC (LTB, *p* < 0.0144; DS-TB, *p* < 0.0001; DR-TB, *p* < 0.0036) (**Figure 2F**).

Regarding active B cells, the frequency of IgG^+^ B cells was increased in DS-TB and DR-TB compared with UC (*p* < 0.0035 and *p* < 0.0375, respectively) (**Figure 2G**), and IgM^+^ B cells were significantly higher in all TB groups than UC (LTB, *p* < 0.0004; DS-TB, *p* < 0.0378; DR-TB, *p* < 0.0025) (**Figure 2H**).

Interestingly, when comparing the frequency of activated B cells (IgM^+^ or IgG^+^), our results showed an elevated IgG frequency compared with IgM active B cells in UC subjects (*p* < 0.0207). DS-TB showed the same trend as UC, but a statistical difference was not found. The LTB group showed a higher frequency of IgM active B cells than IgG (*p* < 0.0355). Although DR-TB patients showed a higher frequency of IgM active B cells than IgG, we did not find a statistical difference (**Figure S2A**).

Finally, visualization of the active B-cell subsets on FlowSOM also evidenced that IgM^+^ or IgG^+^ expresses CD38; there was no difference in the proportion of IgG^+^CD38^+^ B cells, but IgM^+^CD38^+^ cells are increased in LTB (≈2-fold) and decreased in DS-TB (≈2.6-fold) and DR-TB patients (≈1.3-fold) compared with UC (**Figure 2I**). This shows that ATB patients have altered the frequency of peripheral B cells.

### 3.3 LTB and ATB have decreased the frequency of CD19^+^CD20^−^ cells but not plasma B cells

**Figure 3:**
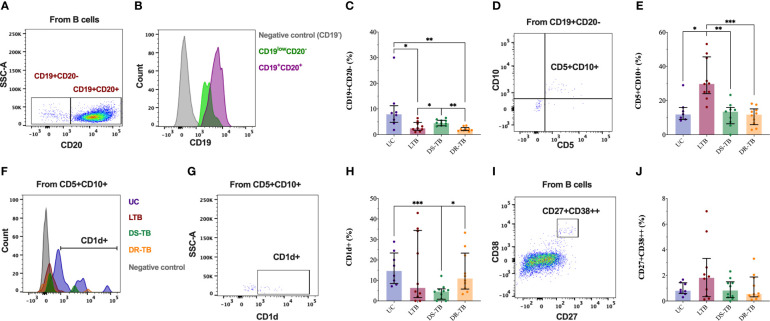
CD19^+^CD20^−^ cells are decreased in the TB groups. Representative flow cytometry plot of HC showing CD20 expression in B cells CD19^+^ **(A)**. Representative histograms of flow cytometry data of CD19 events of HC subjects showing surface marker expression in comparison to CD19 cells in CD19^+^CD20^−^ B cells (green color), B cells (violet color), and non-B cells (gray color) **(B)**. The frequency of plasma cells assessed in the study groups gated by CD20 and CD19 markers **(C)**. The representative dot plot of LTB patients shows co-expression of CD10 and CD5 markers in CD19^+^CD20^−^ B cells **(D)**. The frequency of CD5^+^CD10^+^ into CD19^+^CD20^−^ B cells assessed in the study groups gated **(E)**. Representative histograms of flow cytometry data of CD5^+^CD10^+^ into CD19^+^CD20^−^ B cells of one UC (purple), LTB (red), DS-TB (green), DR-TB (orange), and non-B cells (gray) showing the surface expression of CD1d **(F)**. Representative dot plot of CD1d^+^ cells in CD5^+^CD10^+^ plasma cells **(G)**. The frequency of CD1d^+^ cells in CD5^+^CD10^+^ cells was assessed in the study groups gated **(H)**. Representative dot plot of plasma B cells into B cells gated by co-expression of CD27 and CD38 **(I)**. The frequency of plasma cells CD27^+^CD38^+^ assessed in the study groups gated **(J)**. Data are represented as median and IQR values. Statistical comparisons were performed by the Kruskal–Wallis test. *p < 0.05, **p < 0.01, and ***p < 0.001.

The next aim was to verify the frequency of C19^+^CD20^−^ cells, which was evaluated by flow cytometry (**Figure 3**). By flow cytometry, the CD19^+^CD20^−^ cells were selected (**Figure 3A**); this subpopulation has low CD19 expression, as previously reported (17) (**Figure 3B**). UC has a higher frequency of CD19^+^CD20^−^ cells than LTB and DR-TB patients (*p* < 0.0320 and *p* < 0.0011, respectively). Similarly, DS-TB had a high frequency of CD19^+^CD20^−^ cells compared with LTB and DR-TB (*p* < 0.0350 and *p* < 0.0003, respectively) (**Figure 3C**).

CD5 and CD10 expressions were evaluated on the CD19^+^CD20^−^ cells (**Figure 3D**). The percentage of CD5^+^CD10^+^ increased in LTB compared with UC, DS-TB, and DR-TB (*p* < 0.0199, *p* < 0.0038, and *p* < 0.0008, respectively) (**Figure 3E**). Moreover, CD1d expression was also evaluated in CD5^+^CD10^+^ (**Figures 3F, G**), and DS-TB patients presented a low frequency of CD5^+^CD10^+^CD1d^+^ compared with UC and DR-TB (*p* < 0.0003 and *p* < 0.0113, respectively) (**Figure 3H**). Thus, CD19^+^CD20^−^ cells are less abundant in LTB and DR-TB patients; however, LTB has increased CD19^+^CD20^−^CD5^+^CD10^+^ cells.

The co-expression of CD27 and CD38 was evaluated into the B-cell gate by flow cytometry to identify the frequency of plasma B cells (**Figure 3I**). However, we did not observe significant changes in the frequency of plasma B cells (**Figure 3J**).

### 3.4 DR- and DS-TB have similar immune fingerprinting, which is not shared with LTB

The ATB groups showed an increase of naive B cells, but activated are decreased. So, we decided to quantify the total IgM and IgG serum levels to confirm if the available antibody production is affected in these patients.

**Figure 4:**
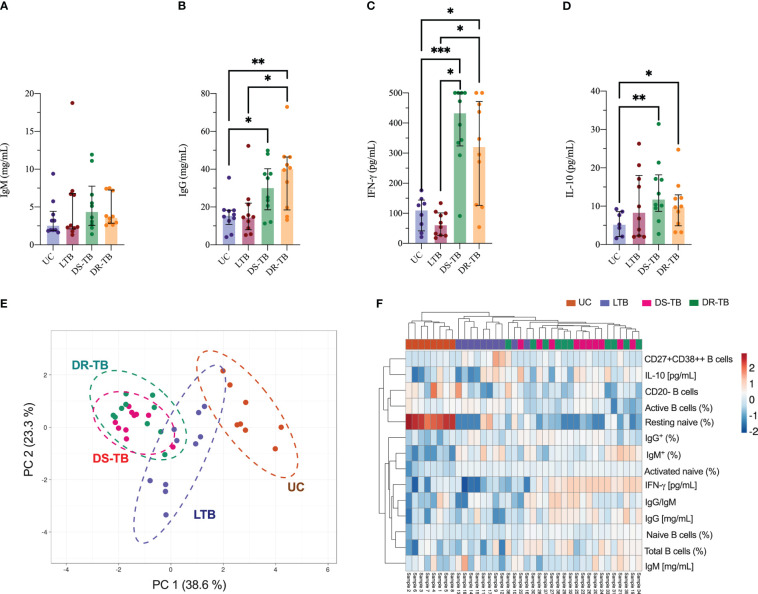
Association between soluble factors and phenotype B cells in TB groups. Quantitative analysis of total serum IgM **(A)** and IgG **(B)** immunoglobulins, IFN-γ **(C)**, and IL-10 **(D)** plasma levels. PCA **(E)** and heatmap plot **(F)** were generated with soluble factors and phenotype B-cell data of each sample. Data are represented as median and IQR values. Statistical comparisons were performed by the Kruskal-Wallis test. *p < 0.05, **p < 0.01, and ***p < 0.001.

Our data showed that IgM is not different between groups (**Figure 4A**), but DS-TB and DR-TB have higher levels of IgG than UC (*p* < 0.0232 and *p* < 0.0445, respectively), and DR-TB has even higher levels of IgG than LTB (*p* < 0.0185) (**Figure 4B**). Although the IgM/IgG ratios reflected a trend to less ratio in DR-TB patients, it was not statistically different among the HC and TB groups (**Figure S2B**).

To verify if the alteration of B-cell subsets hinders the Th1/Th2 polarization as previously reported, IFN-γ and IL-10 were quantified in plasma. DS-TB and DR-TB showed higher IFN-γ levels than UC (*p* < 0.0008 and *p* < 0.0152, respectively) and LTB (*p* < 0.0010 and *p* < 0.0186, respectively) (**Figure 4C**). Regarding IL-10 levels, the ATB groups have higher levels than UC (*p* < 0.0068 and *p* < 0.0459, respectively) but not LTB (**Figure 4D**).

Using a PCA analysis, we identified if one of the TB statuses induces a specific immune fingerprinting. Data showed that DR-TB displays a similar fingerprinting to DS-TB characterized by a positive correlation between IFN-γ (pg/mL), IL-10 (pg/mL), total B cells (%), IgG (mg/mL), and ratio IgG/IgM; this profile separates the ATB group from LTB and UC (**Figure 4E**, green dotted line).

Measurements made in this study suggest that DR-TB and DS-TB are groups that share diverse characteristics, which makes it difficult to divide them (**Figure 4E**, dotted green and purple lines, respectively); both groups showed a negative correlation between IFN-γ (mg/mL) and resting naive B cells (%). The LTB group shared a profile with the UC group (**Figure 4E**, dotted brown line). These results were confirmed and plotted as a heatmap (**Figure 4F**), where we observed that a similar immune fingerprinting is shown by DS-TB and DR-TB using a subset of B cells, immunoglobulins, and cytokines.

### 3.5 Alteration of B-cell subsets correlates with increased IL-10 after weight loss in TB groups

Information about potential correlations among B-cell subpopulations and hematological and biochemical parameters is limited. First, a global analysis of all parameters was performed to identify correlations (**Figure S3**). Thereafter, correlations more specific among B-cell subsets (identified as “modify” in this study) and clinical parameters were evaluated to explore the possibility of using B-cell subsets as a marker of prognosis in the TB spectrum. **Figure S4A** shows 37 positive correlations and 43 negative when TB groups are considered as one group. Then, we took the stronger correlations from **Figure S4A**, where B-cell subsets are involved, and we observed that B cells correlated negatively with hematocrit (**Figure S4B**) and with BMI (**Figure S4C**) while correlated positively with neutrophils (**Figure S4D**).

**Figure 5:**
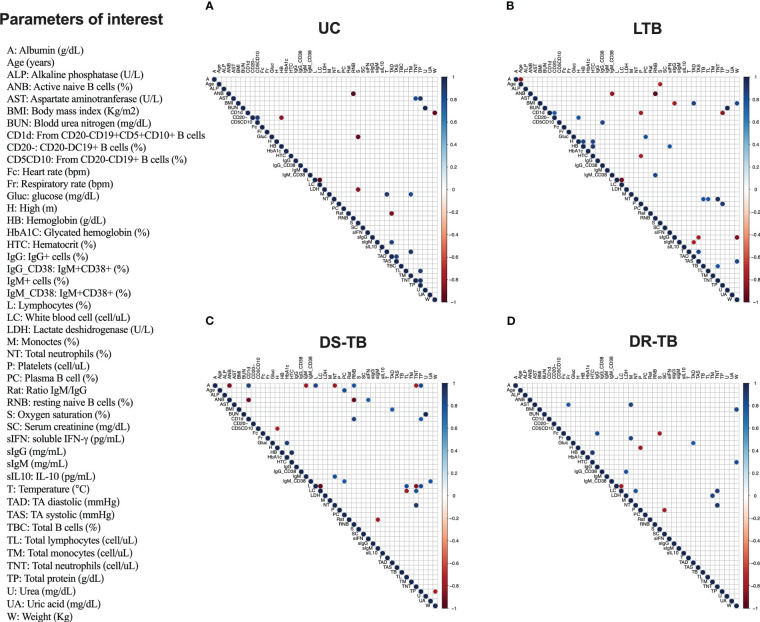
Correlation matrix of demographic and clinical characteristics with serum-derived proteins and frequency of B cell subsets by TB groups. Correlation matrix of demographic and clinical parameters with serum-derived proteins and frequency of B cell subsets in uninfected contacts (UC) **(A)**, latent TB subjects (LTB) **(B)**, drug-susceptible M.tb (DS-TB) **(C)**, and rifampicin-resistant (DR-TB) **(D)**. Only significantly correlated (*p* ≤ 0.01) mediator interactions are shown. The correlogram shows positive and negative correlations in blue and red, respectively. The size and color intensity of the dots are proportional to the Spearman correlation coefficients (*r*_s_).

However, when TB groups were individually evaluated, we observed that UC has positive correlations between CD19^+^CD20^−^ B cells and CD19^+^CD20^−^CD5^+^CD10^+^ B cells (*r*_s_ = 0.90; *p* < 0.0024) and soluble IgM and TA diastolic (*r*_s_ = 0.85; *p* < 0.0045). Negative correlations were observed between activated naive B cells (ANBs) and resting naive B cells (RNBs) (*r*_s_ = −1.0; *p* < 0.00005), CD19^+^CD20^−^ B cells and hemoglobin (r_s_ = -0.74; *p* < 0.007), CD19^+^CD20^−^CD5^+^CD10^+^CD1d^+^ B cells and weight (*r*_s_ = −0.92; *p* < 0.0011), and ratio IgM/IgG and TA diastolic (*r*_s_ = −0.89; *p* < 0.0024) (**Figure 5A**).

LTB showed positive correlations between total B cells with neutrophil count (*r*_s_ = 0.80; *p* < 0.00082), IgG^+^CD38 active with CD19^+^CD20^−^CD5^+^CD10^+^ B cells (*r*_s_ = 0.70; *p* < 0.0014), and RNB with IgM^+^CD38^+^ active B cells (*r*_s_ = 0.85; *p* < 0.0035), and CD19^+^CD20^−^ B cells had two correlations: CD19^+^CD20^−^CD5^+^CD10^+^CD1d^+^ B cells (*r*_s_ = 0.75; *p* < 0.0097) and height (*r*_s_ = 0.78; *p* < 0.0072) and plasma B cell and glucose (*r*_s_ = 0.80; *p* < 0.005) (**Figure 5B**). Moreover, negative correlations were observed between soluble IgG and weight (*r*_s_ = −0.89; *p* < 0.0014), TA systolic (*r*_s_ = −0.78; *p* < 0.007), and BMI (*r*_s_ = −0.81; *p* < 0.008). ANB had two correlations: with RNB (*r*_s_ = −1.0; *p* < 0.0000001) and IgM^+^CD38^+^ activated B cells (*r*_s_ = −0.85; *p* < 0.0035). Soluble IgM correlated with TA diastolic (*r*_s_ = −0.77; *p* < 0.01), and CD19^+^CD20^−^CD5^+^CD10^+^CD1d^+^ B cells correlated with two parameters: platelets (*r*_s_ = −0.8; *p* < 0.005) and total protein (*r*_s_ = −0.85; *p* < 0.0018) (**Figure 5B**).

DS-TB showed a great diversity of correlations; among positives, CD19^+^CD20^−^CD5^+^CD10^+^CD1d^+^ B cells had two correlations: RNB (*r*_s_ = 0.88; *p* < 0.0074) and total protein (*r*_s_ = 0.78; *p* < 0.0072). Albumin levels correlated with RNB (*r*_s_ = 0.79; *p* < 0.0098) and CD19^+^CD20^−^CD5^+^CD10^+^CD1d^+^ B cells (*r*_s_ = 0.87; *p* < 0.0009). IgM^+^CD38^+^ activated B cells correlated with uric acid (*r*_s_ = 0.78; *p* < 0.0072) and with plasma B cells (*r*_s_ = 0.78; *p* < 0.0080). Plasma B cells correlated with age (*r*_s_ = 0.82; *p* < 0.0032). The activated naive B cells showed a correlation with soluble IFN-γ level (*r*_s_ = 0.81; *p* < 0.0041), and IgM^+^ activated B cells correlated with neutrophil count (*r*_s_ = 0.82; *p* < 0.0068). Regarding negative correlations, ANB showed correlations with RNB (*r*_s_ = −1.0; *p* < 0.000001), CD19^+^CD20^−^CD5^+^CD10^+^CD1d^+^ B cells (*r*_s_ = −0.93; *p* < 0.00011), and albumin (*r*_s_ = 0.79; *p* < 0.00098). Meanwhile, albumin showed a correlation with two parameters: IgM^+^ activated B cells (*r*_s_ = −0.82; *p* < 0.0068) and neutrophil count (*r*_s_ = −0.82; *p* < 0.0068). The IgM/IgG ratio showed a correlation with soluble IgM level (*r*_s_ = −0.81; *p* < 0.0082), and finally, CD19^+^CD20^−^CD5^+^CD10^+^ B cells showed a correlation with lactate dehydrogenase (*r*_s_ = −0.79; *p* < 0.0098) (**Figure 5C**).

DR-TB only showed positive correlations among the IgG^+^CD38^+^ active B cells and lactate dehydrogenase (LDH) (*r*_s_ = 0.81; *p* < 0.0051) and IgG^+^ active B cells and heart rate (Fc) (*r*_s_ = 0.82; *p* < 0.0068) (**Figure 5D**). We did not observe negative correlations between B-cell subsets, immunoglobulins, and clinical parameters.

Together, these correlations support the hypothesis that B cells favor neutrophil recruitment and, at least in DS-TB and DR-TB, help maintain an inflammatory status characterized by a high level of IFN-γ.

### 3.6 B cells of DR-TB patients have reduced capacity to produce IFN-γ or IL-10 as a response to *M.tb* antigens

PBMCs were cultured and stimulated with total proteins (TPs) and lipids (TLs) of *M.tb* to evaluate the capacity of B cells to produce IFN-γ and IL-10 (**Figure S5A**).

**Figure 6:**
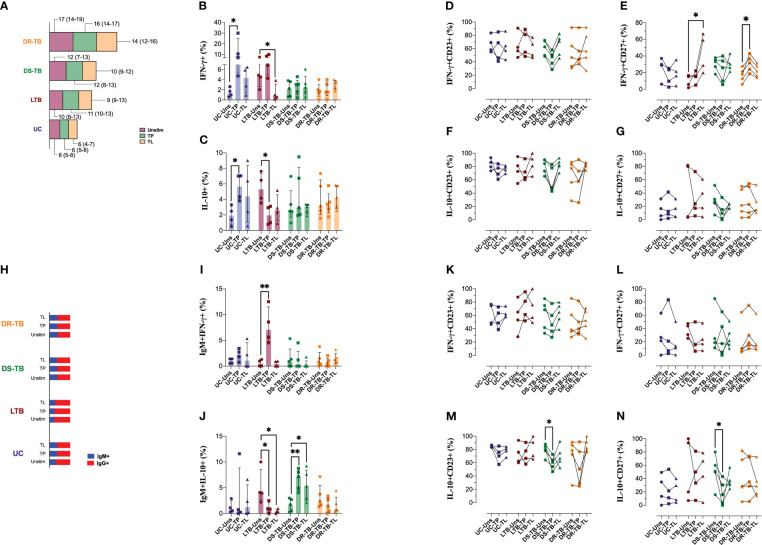
Distinct patterns in the stimulation-induced IFN-γ and IL-10 production of B cells in individuals with TB. PBMCs from the four groups, UC, LTB, DS-TB, and DR-TB, were stimulated for 3 days with total proteins (TPs) or lipids (TLs) of *M.tb*. Unstimulated condition was included as a control stimulation (Unstim). Frequency of B cells assessed in the study groups gated by the CD19 marker **(A)**. The frequency of cytokine-positive cells out of CD19^+^ B cells for each stimulation was analyzed to IFN-γ **(B)** and IL-10 **(C)**. The frequency of IFN-γ^+^ B cells for each condition was analyzed to CD23 **(D)** or CD27 **(E)**. The frequency of IL-10^+^ B cells for each condition was analyzed to CD23 **(F)** or CD27 **(G)**. Analysis of IgM^+^ frequencies **(H)**. The frequency of cytokine-positive cells out of IgM^+^ cells for each stimulation was analyzed to IFN-γ **(I)** and IL-10 **(J)**. The frequency of IFN-γ^+^IgM^+^ cells for each condition was analyzed to CD23 **(K)** or CD27 **(L)**. The frequency of IL-10^+^IgM^+^ cells for each condition was analyzed to CD23 **(M)** or CD27 **(N)**. Data are represented as median and IQR values. Statistical comparisons were performed by the Kruskal–Wallis test. *p < 0.05 and **p < 0.01.

In concordance with **Figure 2A**, DR-TB has a high frequency of total B cells, even unstimulated or under the stimulus, compared with the other groups (**Figure 6A**). Data showed that UC increased the frequency of IFN-γ-producing B cells in response to TP stimulation (*p* < 0.0162) but not with TL. LTB showed a high proportion of IFN-γ-producing B cells at baseline, and it was not modified with TP; however, TL stimulus decreased this frequency (*p* < 0.0286) (**Figure 6B**). Regarding IL-10-producing B cells, TP increased IL-10-producing B cells in UC (*p* < 0.0286), whereas TP decreased this frequency in LTB (*p* < 0.0482) (**Figure 6C**). For the ATB groups, the frequency of B-cell producers of IFN-γ and IL-10 was not modified even under TP or TL stimuli (**Figures 6B, C**), suggesting that during ATB, the B cells remain in a state of tolerance to *M.tb* antigens.

CD23 and CD27 expression on B cells showed that both TP or TL did not modify the frequency of CD23^+^ and CD27^+^ B cells (**Figures S5B, C**). We did not find differences regarding CD23 frequency on IFN-γ-producing B cells (**Figure 6D**). However, IFN-γ-producing B cells of LTB increased their CD27 expression when stimulated with TL (*p* < 0.0216), and DR-TB also increased the expression of CD27 on IFN-γ-producing B cells when stimulated with TP (*p* < 0.0473) (**Figure 6E**). CD23 and CD27 expression on IL-10-producing B cells did not modify under stimulation (**Figures 6F, G**).

The proportion of B cells positive to IgM (blue) and IgG (red) is not modified by *M.tb* antigens (**Figure 6H**). Concerning IFN-γ and IL-10 production by IgM and IgG B cells, our data showed that while UC did not change the cytokine production, LTB increased the IFN-γ production with TP stimulus (*p* < 0.0017) (**Figure 6I**) and decreased IL-10 with TP (*p* < 0.0414) and TL (*p* < 0.0208). DS-TB increased IL-10-producing IgM^+^ B cells when boosted with TP (*p* < 0.0076) and TL (*p* < 0.0319). DR-TB patients did not show changes (**Figure 6J**).

The number of circulating IgM^+^CD23^+^ B cells was significantly reduced in UC when they were stimulated with TP (*p* < 0.0285) and increased in DR-TB under the same stimulus (*p* < 0.0324) (**Figure S5D**). As previously described on total B cells, CD27 frequency did not change in IgM^+^ B cells (**Figure S5E**). The frequency of IFN-γ-producing IgM^+^ B cells and positive to CD23 or CD27 was not modified after stimuli with *M.tb* antigens (**Figures 6K, L**, respectively). Contrary, the frequency of IL-10-producing IgM^+^ B cells and positive to CD23 (*p* < 0.0324) or CD27 (*p* < 0.0324) was decreased in DS-TB when they received the TP stimulus (**Figures 6M, N**, respectively).

In summary, *M.tb* TP or TL stimulation led to a polyfunctional cytokine response associated with production from IgM^+^ B cells and redistribution of CD23^+^ or CD27^+^ subsets in the TB context.

## 4 Discussion

Our study has revealed differences in the redistribution of B cells, circulatory cytokines, and antibodies in DR-TB patients compared with those in UC, LTB, and DS-TB. We have also observed that *M.tb*’s protein (TP) or lipid (TL) stimuli can elicit varying responses from B cells depending on the infection status. These responses involve modification of the B-cell subsets and the production of IFN-γ or IL-10. These findings indicate that the B-cell compartment plays a crucial role during TB and highlights its immunomodulatory function in the immune response to *M.tb*.

B cells in peripheral blood can be identified based on various markers that indicate their lineage and differentiation, such as naive, activated, plasma, and memory B cells (17). Reports suggested that B-cell subset frequencies are modified during chronic inflammation by both virus and bacteria, including *Mycobacterium* (23–25). While it has been confirmed that B cells promote the control of *M.tb* in lymphoid follicles during ATB infection, modulating the Th1 immunity by T cells (4, 26, 27), their function is often considered secondary. However, B cells may enhance their activation and differentiation profile in peripheral blood before reaching the lung lymphoid follicles.

Based on our research, we have described that the levels of B cells in the bloodstream of individuals with active TB (DS- and DR-TB) and those with LTB are comparable to those found in uninfected contacts. This finding is consistent with previous research exploring individuals recently diagnosed and naive to treatment (28, 29).

Our research findings revealed that patients diagnosed with DR-TB have increased B-cell frequency in their peripheral blood. This point is particularly intriguing; although there is limited research on the *in-vitro* and *in-vivo* pathogenesis and adaptation of DR-TB strains, we hypothesized that the phenotypic and genotypic changes in *M.tb* that lead to resistance, including alterations in cell envelope components and intrinsic and extrinsic factors, are responsible for increasing the immunological cell demand needed to clear the infection.

Our naive and active B-cell data corroborate previous studies that described those frequencies not differing among control groups and LTB or DS-TB (19); however, in agreement with a previous report (30), we observed high frequencies of naive B cells in TB patients with a dominium of resting naive B cells. We reasoned that the increased naive B-cell frequencies in DR-TB patients would be a demand for emergency lymphopoiesis in a chronic infection context where it is necessary to have more antigen-inexperienced mature cells as has been previously associated with *M.tb* infection (31).

After the activation process, the B cells can remodel stromal structures and limit the activation of T cells in the lymph nodes (4). Our study showed that activated B cells decreased in DR-TB patients; therefore, our data could reflect the migration process in the chronic context of TB in DR-TB patients. Abreu MT et al. reported that drug-resistant TB patients have a low frequency of unswitched memory B cells and plasma cells in the peripheral blood (30). Here, we also observed a low frequency of plasma cells and active B cells.

It is becoming clear that CD38 has been used extensively to classify various subpopulations of lymphocytes as an activation marker. Several strategies to identify *M.tb*‐specific CD4 T cells were proposed that integrate CD38 as a potential biomarker for ATB (32, 33). However, it has been shown that CD38 can be expressed on B cells as a component of the BCR coreceptor complex, both activated or not activated B cells exerting a modulatory effect on the B-cell activation threshold by controlling CD19 localization (34). CD38 is increased in T cells of TB patients, and CD38 expression on IFN-γ-producing T and B cells can be associated with *M.tb* infection resolution (12, 35). Our work results from the use of CD38 as a potential biomarker in B cells for all TB spectra compared with HC subjects. This comparison shows the frequencies of resting/activated naive B cells and IgM^+^CD38^+^ activated B cells, and the more circulating resting naive B cells are linked to poor B-cell homeostasis as was revealed by the negative correlation with B-cell subpopulations.

It is known that most of the B cells that reach the germinal center of mice exposed to BCG have greater gene expression of CD38 (36); although it was not demonstrated in this work, our results open the possibility of looking for whether the B cells of germinal centers gain expression of CD38 *in situ* or migrate with that phenotype in TB patients. These results highlight the importance of monitoring B-cell activity in DR-TB patients and the potential impact on disease progression.

In agreement with previous studies, we observed that in ATB patients, the circulatory microenvironment is characterized by a high level of IgG and IL-10 (37, 38). DS-TB can be differentiated from UC but shares some characteristics with LTB. In this regard, recently, we reported that LTB presents an increase of CD5^+^ B cells, which can produce IFN-γ, IL-10, and IL-4; in contrast, CD5^+^ B cells from ATB produce mainly anti-inflammatory cytokines as a response to *M.tb* stimulus (39). Thus, B-cell subsets mediate the immune response against *M.tb* not only by the classical function of antibody production; apparently, these subsets are imperative to maintain the pro- or anti-inflammatory status in the TB spectrum.

The memory B-cell proportion shows different compositions across the infection chronicity of TB; for example, ATB subjects have increased atypical memory B cells compared with LTB patients (40). Similar results were found in this work. However, we detailed that even LTB shows a low frequency of memory B cells, like plasma cells in HC subjects, compared with the LTB group. This could be associated with adaptative responses originating from some virulence factors (lipids or proteins) released into the air by ATB patients through exhaled breath condensates (41), which are inhaled by HC subjects. Reports indicated that the PPD-specific IgM and IgG responses are higher in LTB and ATB than in healthy donors (42, 43); however, the knowledge in DR-TB is limited. Future studies should explore this field for potential therapies or biomarkers. Although this study only evaluated total immunoglobulin levels, we propose investigating the total *M.tb*-specific immunoglobulin levels in the future and exploring differences in the humoral responses exhibited by LTB, DS-TB, and DR-TB in more detail.

We hypothesized that the frequency of plasma B cells in DR-TB patients could deteriorate the adaptative responses through antibody responses because their increase is associated with survival and elimination of infectious diseases (44). However, we did not find differences between groups. In contrast, a previous report that indicated an increase in plasma cell responses in early infection reduced in latency and DR-TB but increased during ATB (45). The differences may be associated with the timing of sampling, processing, or number of events acquired. Here, we reported for the first time that CD19^+^CD20^−^ B cells can acquire CD5 and CD10 co-expression in the TB context, suggesting that *M.tb* favors the modulation of cells with the regulatory phenotype; however, this study has the limitation of not evaluating the regulatory B cells with the phenotype previously defined (46, 47). To draw some definitive conclusions in this regard, developing a study with a sufficiently large cohort over a follow-up period of several years is required to explore regulatory B-cell phenotypes.

Diverse studies have demonstrated the relevance of the increase in total B cells after weight loss. We observed that ATB presents high IgG-soluble levels. Considering that the weight loss induced reduced IgG N-glycosylation, resulting in reduced glycan and biological activity (48), our result opens a new question regarding the functional activity antibodies maintained in ATB in these conditions that can reduce efficacy in the available action of antibodies against *M.tb*.

Differential glycosylation states on the Fc domain were observed between LTB and ATB (49). However, based on the observations in this phenomenon, it has been postulated that increased galactosylation correlates with reduced antibody efficacy during active TB and could represent the expansion of non-specific plasma cells, which poorly target *M.tb* (50). This result contrasts with the reduced IgG antibody production but elevated plasma cells that, in agreement with previous results, could have high functional properties against *M.tb* (51).

To confirm the capacity of B-lymphocyte subsets to respond to *M.tb* antigens, we stimulated PBMCs with TP and TL from *M.tb*, and data showed that during ATB, B cells maintained a hyporesponsive state, which is more prominent during DR-TB. In contrast, LTB subjects present a stimulated state characterized by IFN-γ that decreases in the presence of lipids of *M.tb* and IL-10 basal production that is diminished in the presence of both proteins and lipid stimulus. In contrast to the total B-cell results, IgM^+^ B cells displayed a markedly different response, mainly by inducing IFN-γ in LTB subjects, and exhibited high IL-10 levels in DS-TB patients when stimulated. Together, it is suggested that restimulations to *M.tb* antigens could reduce responses to stimulation of B cells, as previously observed in other circulatory leukocytes (9). We did not identify which antigens in total protein and lipids were responsible for the immune responses presented in this study. However, these results could prove useful in clinical testing where there is interest in searching for new biomarkers; the high level of details presented here may be helpful to understand better how B cells react with *M.tb*. Some reports indicate that autocrine IL-10 promotes B-cell differentiation into plasmablast (52). We observed that *in-vitro* IgM^+^ B cells produce high levels of IL-10 in response to TP and TL in DS-TB patients. We consider that during ATB, continuous levels of stimulation maintain a pool of plasma cells.

## 5 Conclusions

**Figure 7:**
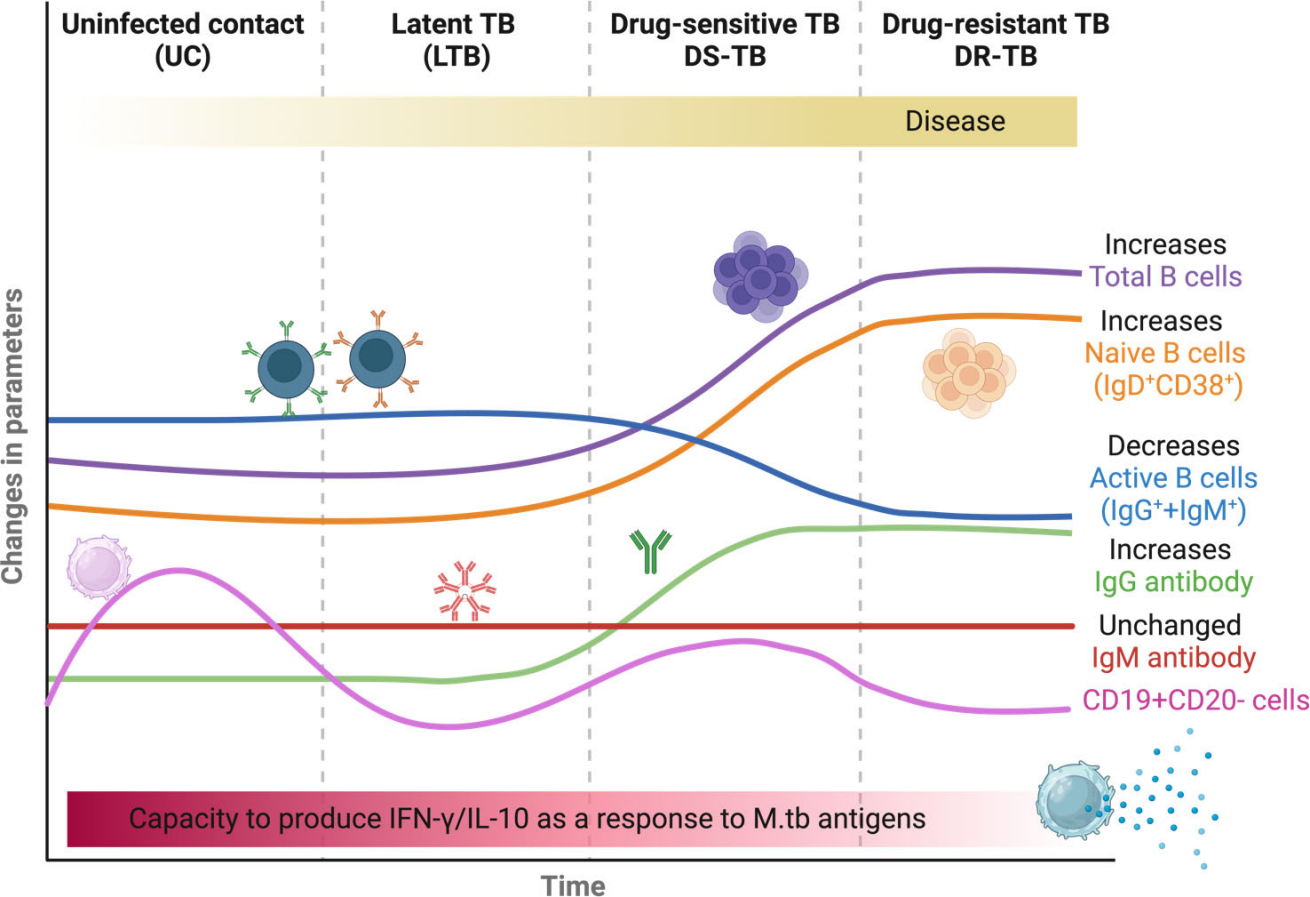
Summary of the presumptive B-cell fingerprinting between TB groups. The figure was created in BioRender.

This study highlights the differences between TB patients’ B-lymphocyte profiles (**Figure 7**). According to the B-cell phenotypes evaluated here, DS-TB and DR-TB share an immune fingerprint, which suggests that some of the characteristics being assessed, such as elevated frequency of total B cells, elevated IgG antibody levels, elevated plasma levels of IFN-γ and IL-10, and relative frequencies of naive and activated B cells, could serve as indicators of TB progression and as players in the pro-inflammatory and anti-inflammatory status of TB patients. *In-vitro* cultures suggest that B cells decreased their capability to produce IFN-γ and IL-10 according to TB status. This study also supports the idea that elicitation of adaptative immunity regulated by B cells could be an essential goal for a vaccine and for diagnostic monitoring against TB.

## Data availability statement

The original contributions presented in the study are included in the article/**Supplementary Material**, further inquiries can be directed to the corresponding author/s.

## Ethics statement

The studies involving humans were approved by the Institutional Ethics Committee of the Instituto Nacional de Enfermedades Respiratorias Ismael Cosío Villegas (Protocols numbers B04-15 and B01-22) at Mexico City.. The studies were conducted in accordance with the local legislation and institutional requirements. The participants provided their written informed consent to participate in this study.

## Author contributions

JF-G: Conceptualization, Formal Analysis, Investigation, Methodology, Writing – original draft. AU-S: Methodology, Software, Writing – original draft. LR-L: Formal Analysis, Methodology, Writing – original draft. JC-D: Investigation, Writing – original draft. AC-R: Investigation, Writing – original draft. EC-Q: Investigation, Writing – original draft. RH-P: Investigation, Writing – original draft. LC-G: Conceptualization, Formal Analysis, Investigation, Resources, Supervision, Writing – review & editing.
