# Supplementary material for: Active tuberculosis patients have high systemic IgG levels and B-cell fingerprinting, characterized by a reduced capacity to produce IFN-γ or IL-10 as a response to M.tb antigens
Source: Front Immunol. 2023 Oct 26;14:1263458. doi: 10.3389/fimmu.2023.1263458 (PMC10643169; doi:10.3389/fimmu.2023.1263458)
Supplement: Supplementary file 1 [file DataSheet_1.docx]

**Supplementary Material**

Active tuberculosis patients have B cells-fingerprinting characterized by enhanced IFN-γ, IL-10, and antibody production.

Julio Flores-Gonzalez^1,2^, Alexia Urbán-Solano^1^, Lucero A. Ramón-Luing^1^, Juan Carlos Cancino-Diaz^2^, Araceli Contreras-Rodriguez^2^, Everardo Curiel-Quesada^3^, Rogelio Hernández-Pando^4^, Leslie Chavez-Galan^1*^

^1^Laboratory of Integrative Immunology, Instituto Nacional de Enfermedades Respiratorias Ismael Cosio Villegas, Mexico City, Mexico

^2^Laboratory of Immunomicrobiology, Escuela Nacional de Ciencias Biológicas, Instituto Politécnico Nacional, Department of Microbiology, Mexico City, Mexico

^3^Department of Biochemistry, Escuela Nacional de Ciencias Biológicas, Instituto Politécnico Nacional, Department of Microbiology, Mexico City, Mexico

^4^Section of Experimental Pathology, Instituto Nacional de Ciencias Médicas y Nutrición Salvador Zubirán, Department of Pathology, Mexico City, Mexico

*** Correspondence:**Leslie Chavez-Galan
[lchavezgalan@gmail.com](mailto:lchavezgalan@gmail.com), [lchavez_galan@iner.gob.mx](mailto:lchavez_galan@iner.gob.mx)

Supplementary Figures and Tables

Supplementary Figures


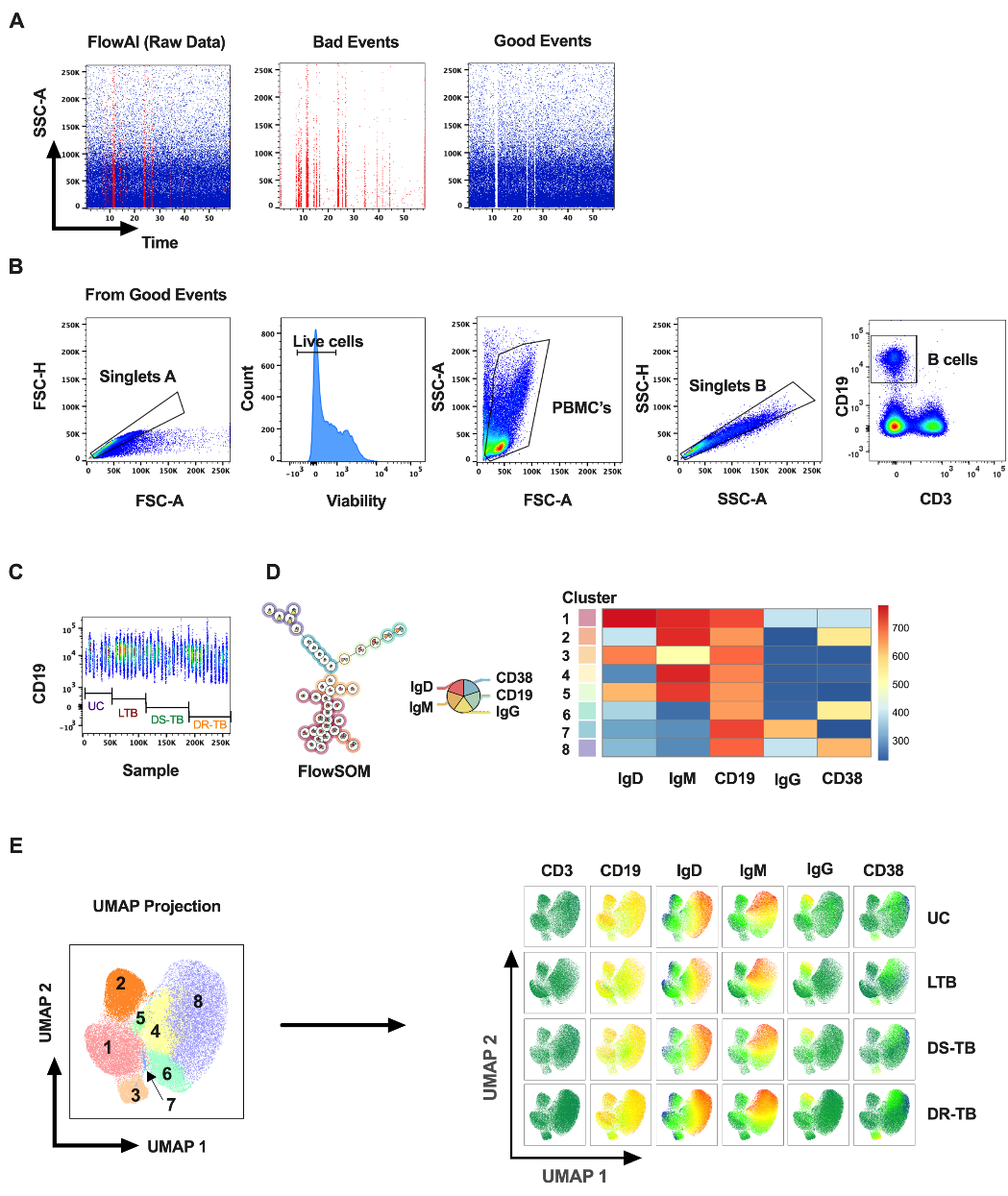


**Supplementary Figure 1.** **General strategy of FlowSOM combined with UMAP for flow cytometry data analysis.** The FlowAI analysis was done as a quality control analysis to detect and remove anomalies from FCS data analyzing the flow rate, signal acquisition, and dynamic range in each sample acquired before analysis (**A**). From good events identified in FlowAI analysis, single events identified by forward (FSC-A vs. FSC-H) was made before to analyzed viability. PBMCs was selected trough forward scatter (FSC) and side scatter (SSC) dot plot. A second single events identified by side scatter (FSC-A vs. FSC-H) was made to identify B lymphocytes based on CD19 and CD3 expression (**B**). Concatenate data was made from all FSC CD19-positive data (**C**). FlowsSOM tree fro the B cells subsets based on CD19, IgD, IgM, IgG, and CD38 expression (**D**). UMAP dimensionality reduction performed the whole B cell population grouped cells into four islands and eight groups when a FlowSOM projection was made as clustering channels (**E**).


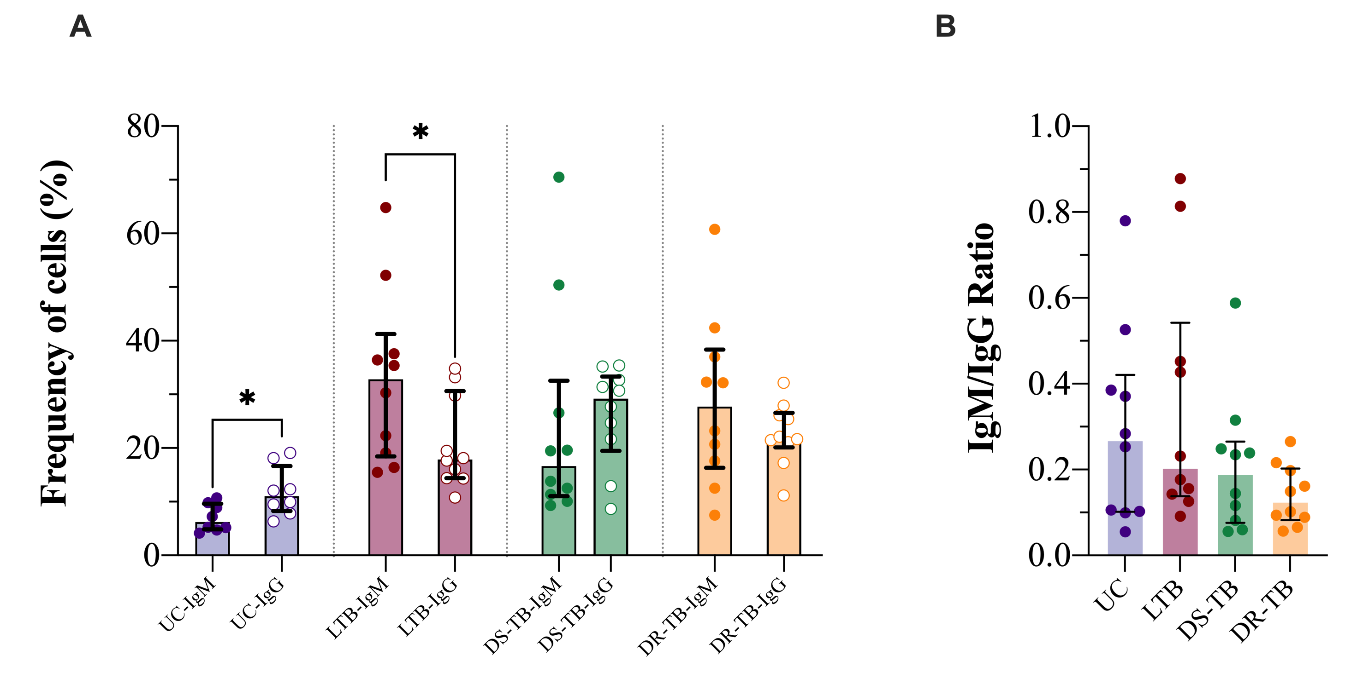


**Supplementary Figure 2.** **Comparative frequency of IgM versus IgG active B lymphocytes.** Frequency of IgM and IgG active B lymphocytes assessed in the study (A). Based on total immunoglobulins in plasma the IgM/IgG ratio was calculated (B). Data are represented as median and IQR values. Statistical comparisons were performed by the Mann–Whitney U test, * p < 0.05.


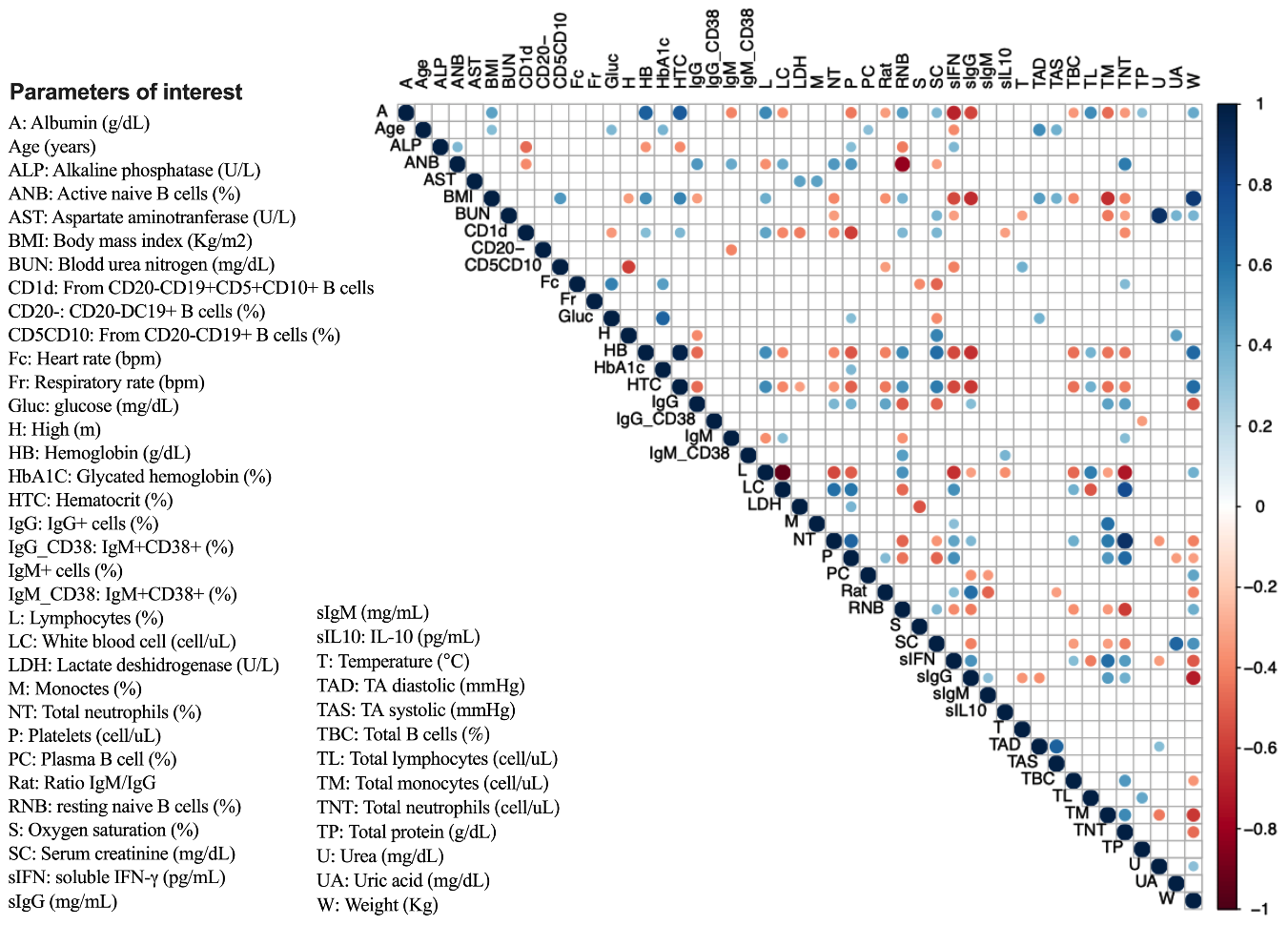


**Supplementary Figure 3.** **Correlation matrix of demographic and clinical characteristics with serum-derived proteins and frequency of B cell subsets.** Correlation matrix of demographic and clinical parameters with serum-derived proteins and frequency of B cell subsets. Only significantly correlated (*p*≤0.05) mediator interactions are shown. In correlogram, positive and negative correlations are shown in blue and red, respectively. The size and color intensity of the dots are proportional to the Spearman correlation coefficients (r_S_).

**
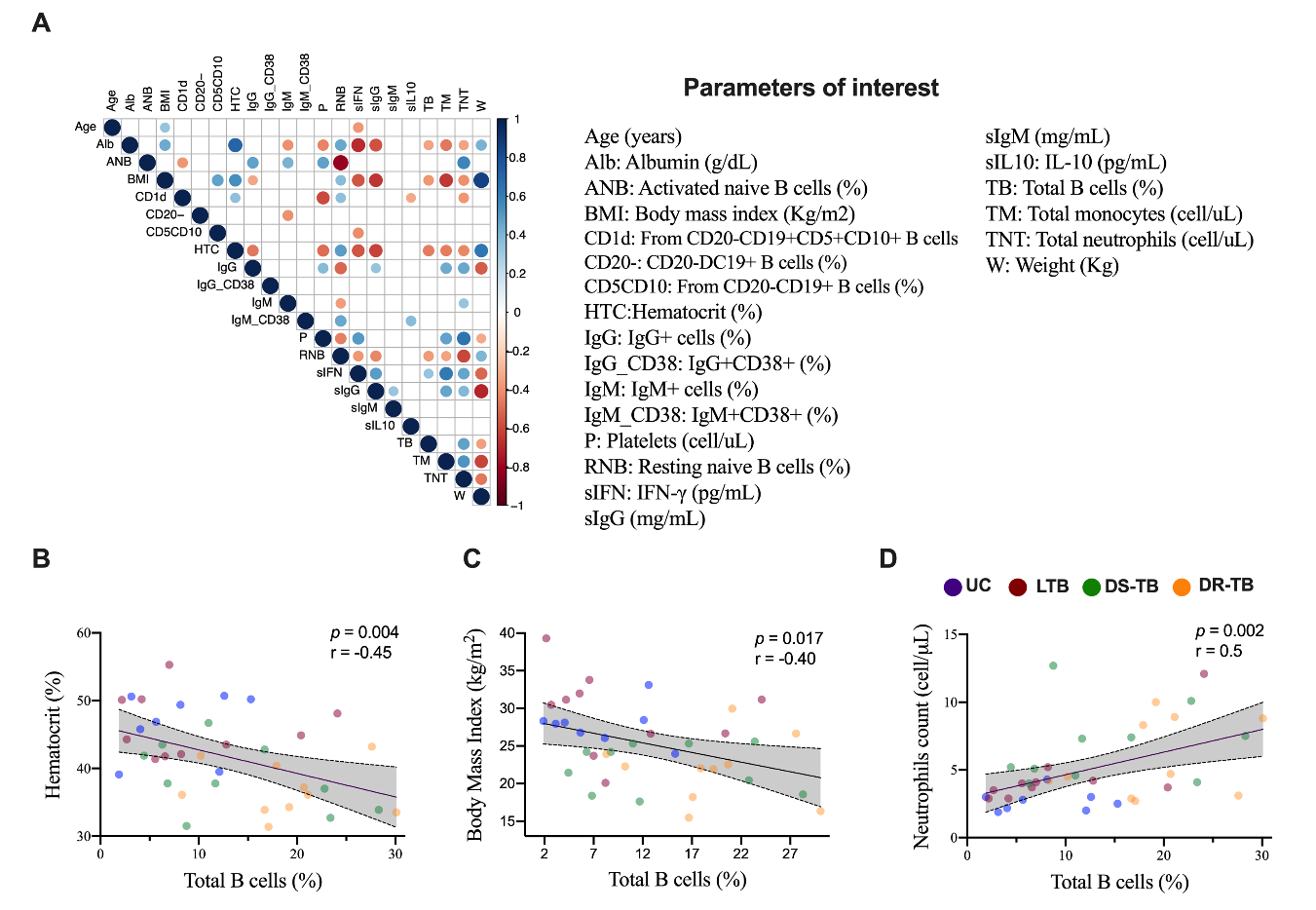
**

**Supplementary Figure 4. Correlogram and correlation analyses in TB groups.** Correlation matrix of parameters of interest with serum-derived proteins and frequency of B cell subsets, and demographic and clinical characteristics included (**A**). Correlation plots between Total B cells (%) vs hematocrit (%) (**B**), vs BMI (**C**), and vs Neutrophil count (cell/μL) (**D**). Only significantly correlated (P ≤ 0.05) mediator interactions are shown. In correlogram, positive and negative correlations are shown in blue and red, respectively. The size and color intensity of the dots are proportional to the Spearman correlation coefficients (r_S_). In correlation plots the regression show as black line (with 95% confidence intervals).


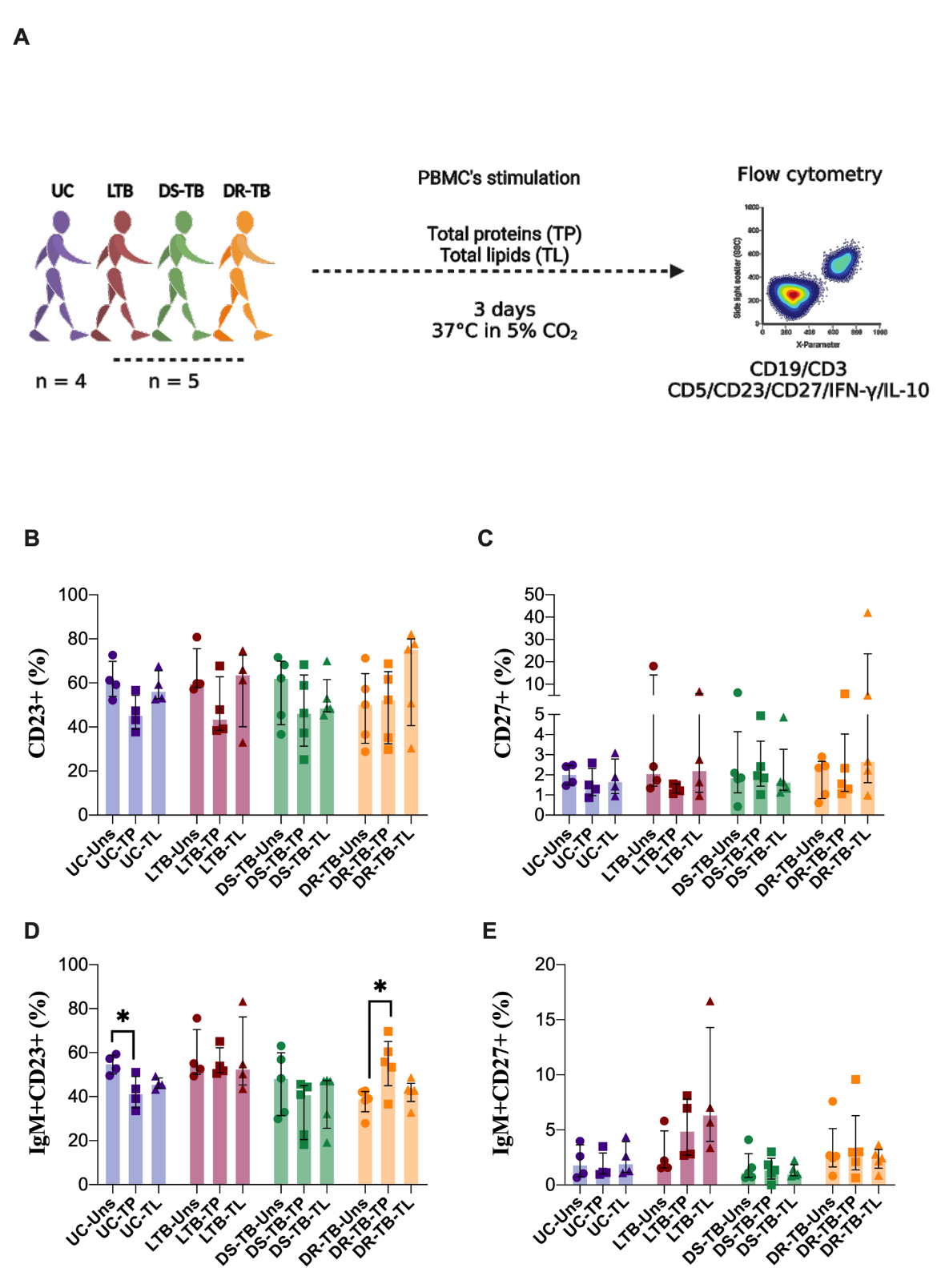


**Supplementary Figure 5.** **Comparative frequency of IgM versus IgG active B lymphocytes.** PBMCs from four groups, including HC, LTB and DS-TB and DR-TB were stimulated for 3 days with total proteins (TP) or lipids (TL) of Mtb. Unstimulated condition was included as a control stimulation (Unstim). Cells were analyzed by flow cytometry (A). Analysis of CD23+ and CD27+ B cells frequencies (B and C). Analysis of IgM+CD23+ and IgM+CD27+ B cells frequencies (D and E). Data are represented as median and IQR values. Statistical comparisons were performed by Kruskal-Wallis’s test, * *p* < 0.05.

Supplementary Tables

| **Table S1. Antibodies used for flow cytometry and ELISA**. | | | |
| --- | --- | --- | --- |
| **Antibody** | **Conjugate to** | **Clone/Catalog^1^** | **Company** |
| CD19 | BV421 | HIB19 | Biolegend |
| CD19 | APC-Cy7 | HIB19 | Biolegend |
| IgD | PE-Cy7 | IA6-2 | Biolegend |
| IgM | PE | MHM88 | Biolegend |
| IgG | FITC | M1310G05 | Biolegend |
| CD3 | APC-Cy7 | OKT3 | Biolegend |
| CD3 | PB | OKT3 | Biolegend |
| CD38 | APC | HIT2 | Biolegend |
| CD23 | PE-Cy7 | EBVCS-5 | Biolegend |
| CD27 | PerCP-Cy5.5 | M-T271 | Biolegend |
| IFN-γ | APC | 4S.B3 | Biolegend |
| IL-10 | AF-488 | JES3-9D7 | Biolegend |
| CD20 | PE-Cy7 | 2H7 | Biolegend |
| CD5 | BV510 | L17F12 | Biolegend |
| CD10 | FITC | HIT10a | Biolegend |
| CD1d | PE | PE | Biolegend |
| Zombie Red Dye | PE-Texas Red | 423109 | Biolegend |
| IFN-γ | NA | 430104 | Biolegend |
| IL-10 | NA | 430604 | Biolegend |
| Total IgM | NA | 88-50620-22 | Invitrogen |
| Total IgG | NA | 88-50550-22 | Invitrogen |

^1^The clone is indicated for flow cytometry antibodies and the catalog number for ELISA. NA, not applicable.
